# Development and validation of primary health care quality assessment tool

**DOI:** 10.1186/s12913-023-10162-x

**Published:** 2023-10-26

**Authors:** Pouria Farrokhi, Ehsan Zarei, Rafat Bagherzadeh, Behrooz Irannejad, Asgar Aghaei Hashjin

**Affiliations:** 1 Department of Health Management and Economics, School of Public Health, Tehran University of Medical Sciences, Tehran, Iran; 2 Department of Health Service Management, School of Management and Medical Education, Shahid Beheshti University of Medical Sciences, Tehran, Iran; 3 School of Health Management and Information Sciences, Iran University of Medical Sciences, Tehran, Iran

**Keywords:** Service Quality, Primary Health Care, Validity, Reliability, Questionnaire

## Abstract

**Background:**

Assessing the quality of health services gives insights to managers about the status of services delivered by them, especially from the client’s perspective. Although various tools have been developed to measure the quality of primary health care (PHC), no specific tool was found in this field in Iran. Therefore, the present study was conducted to develop and validate the quality assessment tool of PHC in Iran.

**Methods:**

This methodological study was conducted in 2021. In the first step, based on a literature review, an initial questionnaire was designed, and its face validity, content validity, construct validity, and reliability were evaluated. Descriptive tests, Kolmogorov-Smirnov, exploratory factor analysis, Kaiser-Myer-Olkin (KMO), and Cronbach’s alpha were performed by using SPSS 22.

**Results:**

The initial questionnaire included 33 items, of which three items were removed due to inconsistency with factorization. The final questionnaire consisted of 30 items and nine dimensions: interaction, efficiency, timeliness, accuracy, consultation, tangibility, safety, accessibility, and environment. The KMO and Cronbach’s alpha index values were 0.734 and 0.82, respectively, indicating acceptable reliability and validity. The developed dimensions represented about 73% of the total variance.

**Conclusion:**

The designed questionnaire has relatively good validity and reliability and can be used to measure the quality of PHC and to identify weaknesses in service delivery. However, researchers should carefully examine it to enhance its applicability as a standard tool for measuring PHC quality.

**Supplementary Information:**

The online version contains supplementary material available at 10.1186/s12913-023-10162-x.

## Background

The World Health Organization (WHO) declared primary health care (PHC) essential for all health systems to provide equitable and efficient services in a 1978 statement in Alma-Ata and a 2008 report in Astana [1, 2]. The robust and affordable PHC systems can deliver satisfactory health outcomes for the populace [3]. Studies in the United States and low- and middle-income countries have also reported that greater access to PHC is associated with improved health and reduced use of costly health care [4, 5]. PHC has been known as one of the necessary foundations for the health-related Sustainable Development Goals (SDGs) and universal health coverage (UHC) [6]. One of the most important policies in the health systems of all countries, including Iran, is to achieve the SDGs, which are now used as a reference in global development guidelines and determine social and environmental factors in health [1]. The SDGs include 17 global goals, the third of which is dedicated to health: “to ensure healthy lives and promote well-being for all ages” [7].

The achievement of SDG3 targets requires systematic and coherent evidence-based measures to strengthen PHC, emphasizing equity, efficiency, and quality [8, 9]. Quality of care is defined as a degree of health care that increases the likelihood of favorable health outcomes for individuals and populations and is consistent with current professional knowledge [10]. During decades of research, many elements have been described for quality, and the most agreed-upon quality services include safety, effectiveness, and people-centeredness. Furthermore, to realize the benefits of quality health care, health services must be timely, equitable, integrated, and efficient [11].

Since 1984, the development of PHC in Iran commenced with the support of the WHO. The PHC team in Iran comprises a family doctor, a midwife, a health technician, and a unique health force known as “Behvarz” for rural areas. Their main tasks include, inter alia, taking care of pregnant mothers, children, and the elderly, preventing communicable and non-communicable diseases, providing services like vaccination to the target population, and cooperating with the family physician. In large villages, there are also rural health centers in addition to health houses [12, 13]. Iran successfully provided PHC in the 1970 and 1980 s based on the population’s needs, and as a result, health indicators improved significantly [14]. Nonetheless, due to changes in the burden of diseases and demand patterns, the PHC system has faced challenges and problems in meeting the population’s current needs. Providing low-quality services in health centers in dealing with communicable diseases, maternal death, infant mortality, and emerging non-communicable diseases has seriously affected Iran’s health system [15, 16].

The WHO has always been concerned with service quality, and health service providers have sought to achieve and maintain a competitive advantage through quality. Service quality measurement can help identify weaknesses and gaps [17]. Most models and tools for measuring health service quality have been adopted from other industries [18]. One of the first and most important models is the Gronroos model (1984), which considered technical and functional quality dimensions of service quality. Furthermore, Parasuraman (1985), known as the gaps model, introduced the SERVQUAL scale to measure service quality. In this model, quality is defined as meeting customer expectations, i.e., smaller gaps between customers’ perceptions and expectations indicate better service quality. Haywood-Farmer model of service quality (1988) comprises physical facilities and processes, behavioral aspects, and professional judgment and focuses on continuous attention to customers’ preferences and expectations [19, 20].

Although various and valuable tools have been designed and used to measure service quality, it should be considered that the nature of health services and the characteristics and expectations of health service clients are different from those of the other markets [21, 22]. Therefore, specific tools should be designed for health care and its settings, such as inpatient, emergency, and primary care. Most tools designed to measure health service quality are related to hospital settings, and primary care has received less attention. PHC and its clients differ from other settings, such as hospitals; therefore, they should be assessed with different instruments. According to a recent systematic review, tools developed to evaluate the quality of primary healthcare have shortcomings in terms of their validity and reliability [23]. Therefore, there is still a need to design reliable and valid tools to assess PHC settings. The primary care system in Iran has its own context and characteristics; therefore, it requires its own specific tools to measure and monitor the provision of quality services. Furthermore, the primary care services in Iran are provided in an integrated system of education and service provision, that is, health centers are affiliated to Medical Universities which are all public centers, and private sectors have a minor role in the provision of primary care [12]. The demographic characteristics of primary care users in Iran are different from other countries, that is, primary care is mostly focused on rural areas, and PHC coverage in cities is not adequate [24].

Moreover, the dominant view in the primary care system in Iran is treatment-oriented rather than prevention-oriented [25]. Primary healthcare services are mostly free of charge in Iran, and in order to overcome cultural barriers, most of the providers are local (such as Behvarz). The payment mechanism for primary care is salary-based and per capita [26]. Finally, communications between patients and doctors and among service providers at different levels are incomplete, weak or insufficient due to the existing referral system [27, 28]. Primary healthcare culture in Iran is eroding mainly due to the lack of knowledge of healthcare providers in healthcare centers [28]. Iran is a multicultural country; distinct cultures may hold distinct attitudes towards health, medical care, and technological advancements and may result in different conceptions of health, disease, medical interventions [29]. For example, some sociocultural barriers (e.g., religious restrictions) prevent women from timely breast and cervical cancer screening [30]. They all have a direct impact on the quality of services, especially the process of providing services, the cost of services, diagnoses, and equipment. As a result, patient’s perception of the quality of PHC services in Iran will be different from patients’ perceptions in other countries thus indigenous service quality assessment tools are required. To the best of our knowledge, there are no appropriate and specific tools available to evaluate the quality of primary healthcare (PHC) in Iran [31–34]. Thus, this study endeavored to develop and validate a questionnaire that can accurately measure the quality of PHC.

## Methods

### Study design

This methodological study was conducted to design a questionnaire in Iran in 2021.

### Participants and the sampling method

The study population included all patients of PHC centers affiliated with Iran University of Medical Sciences (IUMS) in Tehran. Three universities of medical sciences provide health services in Tehran city, and each university has several health networks consisting of PHC centers and hospitals. In this study, three (out of seven) health networks affiliated with IUMS were randomly selected due to budget and time constraints. Then, four PHC centers were selected from each network (n = 12) through simple random sampling. The samples were assigned to each PHC center and selected from each center by systematic random sampling.

Cochran’s formula was used to determine the sample size. Cochran’s formula, introduced by William Cochran in 1931, is one of the most widely used methods for calculating statistical sample size. In this study, the significance level was set at 0.05. Cochran’s formula is as follows (N = 290):

$$n = \frac{{\frac{{{z^2}pq}}{{{d^2}}}}}{{1 + \frac{1}{N}\left[ {\frac{{{z^2}pq}}{{{d^2}}} - 1} \right]}}$$

During two months, 300 questionnaires were distributed 280 of which were filled out by patients when leaving the PHC center. Quality is an abstract concept, and those who have understood the entire process of receiving service can better evaluate tangible and intangible services. Therefore, only individuals who had almost all kinds of services, including a doctor’s visit, were included in the study.

#### Developing the questionnaire

Initially, the researchers reviewed the literature in various databases, such as PubMed, Scopus, and Web of Science, to identify questionnaires for evaluating health service quality. Three instruments were selected as the basis for designing the questionnaire. i.e., HEALTHQUAL, an adapted version of SERVQUAL questionnaire designed for evaluating the quality of health care services [35, 36]; Primary Care Assessment Tool, a questionnaire that examines primary care and focuses on accessibility, availability, and referral system [37]; and a researcher-made framework, which focuses on the provision of quality primary care based on patients’ views in Iran [38]. The instruments were reviewed to extract the most relevant items and attributes for assessing the quality of PHC. Similar items were merged, and duplicates were also removed. Questionnaire items were measured on a 5-point Likert scale (5 = strongly agree, 4 = agree, 3 = not sure, 2 = disagree, and 1 = strongly disagree).

#### Face & content validity

The involvement of experts in content validity is common. Two rounds of the Delphi technique were used by local experts to assess the content validity of the questionnaire. The first draft of the questionnaire was sent to eight experts in the field of health service quality to rate the items in terms of difficulty, grammar, the degree of fit between the items and the stated dimensions, the level of ambiguity, and misconceptions in the interpretation of the items on a three-part scale “irrelevant,“ “low or medium relevant” and “relevant.“ The questionnaire items were modified based on the experts’ recommendations. As for the content validity, the necessity of questions and their appropriate design were confirmed by using the content validity ratio (CVR) and the content validity index (CVI), respectively. The approval bases for CVR and CVI were chosen as 0.59 and 0.79 [39, 40], in that order. Experts’ (N = 8) and patients’ (N = 15) opinions were used for face validity. They were given a questionnaire to comment regarding the clarity of the items.

### Construct validity

To assess the construct validity and determine the dimensions and items in each dimension, exploratory factor analysis (EFA) was used. It is a common technique utilized to retain the most critical factors and remove items with low correlation. Moreover, the scree plot was used to determine the number of factors in EFA. The Kaiser–Meyer–Olkin (KMO) was used to ensure the selected sample’s adequacy in EFA. This index is the output of the Bartlett test, which checks whether the correlation matrix is an identity matrix by testing the null hypothesis. If the correlation matrix is equal, it is inappropriate to identify the structure (model). If the significance level of Bartlett’s test is less than 5%, factor analysis is suitable for identifying the structure because the correlation matrix’s assumption of unity (sameness) is rejected. Also, for the KMO index, values between 0.7 and 0.8 were considered middling, and 0.8 to 0.9 were called meritorious [27]. The factors with eigenvalues equal to or higher than one were retained and selected for interpretation.

### Reliability

Cronbach’s α coefficient was calculated to determine the scale’s internal consistency. According to previous studies, alpha values above 0.9 are excellent, above 0.8 are good, above 0.7 are acceptable, and between 0.5 and 0.7 are considered debatable, weak, or unacceptable [41, 42].

### Data analysis

Descriptive tests, Kolmogorov-Smirnov, exploratory factor analysis, Kaiser-Myer-Olkin and Bartlett, and Cronbach’s α were performed by using SPSS version 22 software. Data analysis was performed in three steps. The primary healthcare quality dimensions and the related items were determined through EFA. The criterion for the number of factors to be rotated was eigenvalues greater than 1, and items with factor loadings lower than 0.4 were excluded. Sample adequacy and normal distribution of multivariate data for extraction of the factors were evaluated by the KMO test and Bartlett’s test of Sphericity. To evaluate the reliability of the retained variables in each factor, Cronbach’s α coefficient was calculated, and the coefficients higher than 0.6 were considered acceptable [43].

## Results

### Demographical findings

In this study, 145 of the study participants were female (51.8%), 167 held a university degree (59.6%), 268 were residents of urban areas (95.7%), 161 were married (57.5%), and 137 people (48.9%) had visited health centers four times or more (Table 1).

**Table 1:** Demographic characteristics of the participants (N = 280)

Variables		Frequency	Percentage	Cumulative percentage
Gender	Male	135	48.2	48.2
	Female	145	51.8	100
Education	No schooling	10	3.5	3.5
	Primary and Secondary school	103	36.8	40.3
	University	167	59.6	100
Residence Area	Urban	268	95.7	95.7
	Rural	12	4.3	100
Marital status	Married	161	57.5	57.5
	Single	119	42.5	100
Rate of center visit	First	95	33.9	33.9
	Second	37	13.2	47.1
	Third	11	3.9	51.1
	Fourth or more	137	48.9	100
Total		280	100	

### Content and face validity

The initial questionnaire consisted of 33 items covering five dimensions, namely Tangibility (questions 1 to 7), accessibility (8 to 12), Effectiveness (13 to 20), Interactions (21 to 27), and Efficiency (28 to 33). Three questions were shifted between dimensions during content validity, and four were modified to confirm face validity. The content validity of the questions was confirmed after checking and revising several questions (CVR = 0.69, CVI = 0.81). One hundred twenty-three participants chose the “totally agree” option: “The doctor and other staff’s conversations were clear and understandable to me.“ Also, 13 people selected the “totally disagree” option for the statement “the health center had sufficient and clean toilets” (Table 2).

**Table 2:** Frequency of questionnaire questions

Items	Strongly agree	Agree	Not sure	Disagree	Strongly disagree
1: The health center was clean and tidy.	60	171	33	16	0
2: The appearance and dress of the employees were neat and orderly.	118	152	8	2	0
3: The amenities of the waiting room (TV, water cooler, chairs, magazines, etc.) were at the optimum level.	28	141	47	60	4
4: This center has advanced equipment and facilities.	29	87	104	56	4
5: The noise and crowding of the environment was not annoying.	65	167	24	21	3
6: The temperature (hot and cold) of the environment was suitable.	82	180	0	12	6
7: The health center had sufficient and clean sanitary facilities.	32	123	79	33	13
8: The hours and days of operation of the health center are such that you can easily visit it anytime.	57	115	73	35	0
9: Access to the health center was easy.	71	90	99	20	0
10: It is possible to receive medical advice from this center offline (e.g., via phone, website, etc.).	23	79	131	43	4
11: If the doctor is absent in the health care center, someone else (such as a nurse) will handle your problem.	21	135	113	11	0
12: When visiting the health center, the staff addressed your problem regardless of demographic characteristics such as citizenship, gender, language, etc.	73	136	65	6	0
13: In this center, a safe and comfortable environment is provided to receive services.	61	153	58	8	0
14: Adequate measures are taken in this center to prevent the spread of infection.	60	127	55	34	4
15: Doctors of this center do not make mistakes in their diagnosis.	32	112	128	8	0
16: Nurses and other employees do not make mistakes in providing services.	34	114	128	4	0
17: Doctors and employees have enough expertise and skills to provide service.	49	145	78	8	0
18: In this center, timely measures and sufficient guidance are provided to relieve the patient from pain.	43	166	67	4	0
19: Besides the visit, the doctor discussed ways to prevent other physical and mental diseases (e.g., diabetes, blood pressure, depression, anxiety, etc.).	94	105	57	20	4
20: I received good advice from the doctor or nurse about a healthy lifestyle (e.g. healthy eating, exercise, etc.).	109	99	37	28	4
21: The behavior of the employees (e.g., receptionist, security guard, and cashier) was good.	101	134	14	29	2
22: Filing the case was easy and completed in the shortest possible time.	102	130	19	29	0
23: The process of paying for the visit was easy and fast.	89	162	14	13	2
24: The doctor and other employees were respectful and polite in providing services.	108	125	23	24	0
25: The doctor and other employees answered my questions completely.	111	142	15	12	0
26: The doctor’s and other employees’ words were clear and understandable.	123	126	23	8	0
27: The doctor and other employees listened and paid enough attention to my words.	109	143	16	12	0
28: I visited the doctor on the expected day and hour.	86	147	24	23	0
29: I did not wait long when I entered the health center to the doctor’s room.	74	140	24	37	5
30: The cost of a doctor’s visit to this center is reasonable.	81	147	48	48	0
31: The service I received at this center was worth paying for.	97	134	45	4	0
32: The doctor and nurse were warned and reminded about the arbitrary use of medicine and other medical services without a prescription.	115	119	38	4	4
33: the doctor and other staff tried their best to avoid wasting time.	88	164	14	14	0

### Construct validity

The value of the KMO index was 0.734, so the model was confirmed, and the data related to the factors influencing the quality of primary health services were suitable for factor analysis. Moreover, Bartlett’s test was significant at P < 0.05 which supports the factorability of the data. Based on Kaiser’s criterion, eigenvalues of more than and close to 1 were chosen to determine the number of factors. The results revealed the presence of 9 factors explaining 73% of the variance (Table 3). At this stage, factor loadings of 0.5 and above were chosen.

**Table 3:** Extraction of factors affecting the quality of primary healthcare services

Factors	Eigenvalues of no rotated factors			Eigenvalues of rotated factors		
	Total	Variance	Cumulative Percentage	Total	Variance	Cumulative Percentage
1	9.22	27.95	27.95	3.65	11.07	11.07
2	2.58	7.83	35.78	3.27	9.93	21
3	2.22	6.74	42.52	2.74	8.30	29.30
4	2.10	6.38	48.91	2.71	8.21	37.52
5	1.81	6.49	55.40	2.30	7.98	45.51
6	1.50	5.56	60.97	2.29	7.96	53.47
7	1.29	4.91	65.88	2.05	7.22	60.69
8	1.24	3.86	69.73	1.93	6.86	67.57
9	1.10	3.42	73.16	1.64	5.58	73.16

Table 4 illustrates the rotated matrix of components for factors affecting the quality of health care services and includes factor loading of each variable in the remaining nine factors after rotation. The higher the absolute value of these coefficients, the more important the relevant factor is in the desired variable’s total change (variance difference). The number of items in the questionnaire was reduced from 33 to 30. Item number 27, “The doctor and other employees listened to my words and paid enough attention,“ was removed due to correlation with two factors. Moreover, items number 7, “The health center had sufficient and clean sanitary facilities,“ and number 12, “When visiting the health center, the staff will take care of your problem regardless of demographic characteristics such as citizenship, gender, language, etc.“ were deleted due to lack of correlation with other factors.

**Table 4:** Factor analysis components matrix after rotation (rotated)

Items No	Factors loading								
	Interactions (Factor 1)	Efficiency (Factor 2)	Timeliness (Factor 3)	Accuracy (Factor 4)	Consultation (Factor 5)	Tangibility (Factor 6)	Safety (Factor 7)	Accessibility (Factor 8)	Environment (Factor 9)
21 24 25 26	0.742 0.738 0.736 0.726								
30 32 31 28		0.728 0.720 0.702 0.553							
23 22 29 33			0.749 0.739 0.694 0.554						
16 15 17				0.866 0.825 0.711					
19 20 18					0.825 0.785 0.534				
4 1 3 2						0.743 0.741 0.598 0.568			
14 13							0.650 0.545		
10 8 11 9								0.790 0.664 0.635 0.533	
5 6									0.743 0.697

Based on the results of EFA, the items with the highest correlation were divided into nine dimensions and named based on their content: Interactions (4 items), Efficiency (4 items), Timeliness (4 items), Accuracy (3 items), Consultation (3 items), Tangibility (4 items), Safety (2 items), accessibility (4 items), and Environment (2 items).

Interactions are defined as “polite behavior of service providers along with answering and understandable explanations to the clients,“ efficiency refers to “the efforts made by providers to prevent the provision of unnecessary and costly services,“ Timeliness is about “trying to reduce waiting time and facilitating the filing and payment processes for clients,“ Accuracy means “the level of expertise and skill of providers to reduce errors,“ Consultation applies to “providing patients with adequate recommendations and suggestions to prevent further problems and enjoy healthy lives.“ Tangibility refers to “the level of cleanliness of the physical space, the appearance of employees, modern facilities and equipment,“ Safety is defined as “providing a safe and infection-free environment,“ accessibility is about “the level of convenience in accessing health care services on different days and hours” and the Environment means “suitable temperature and absence of annoying noise in the service delivery environment.“

### Reliability

The value of Cronbach’s alpha for all dimensions was above 0.7, and the value of Cronbach’s alpha for the whole questionnaire was 0.82 (Table 5). Therefore, our questionnaire has good internal consistency. The final version of the questionnaire is provided as a supplementary file.

**Table 5:** Cronbach’s alpha coefficient values

Factors	Number of items	Cronbach’s alpha
Interactions	4	0.768
Efficiency	4	0.774
Timeliness	4	0.768
Accuracy	3	0.799
Consultation	3	0.801
Tangibility	4	0.802
Safety	2	0.791
Accessibility	4	0.804
Environment	2	0.800
Overall	30	0.820

## Discussion

The current study aimed to develop and evaluate the validity and reliability of a questionnaire for assessing the quality of PHC in Iran. The final questionnaire included 30 items in 9 dimensions of interactions, efficiency, timeliness, accuracy, consultation, tangibility, safety, accessibility, and environment. Rezapour et al. introduced a framework for PHC quality with seven dimensions, three of which, access, safety, and efficiency, were aligned with our results [38]. To improve PHC quality, Ogaji et al. developed a Patient Evaluation Scale including items on facilities, length of waiting time, staff manner, and safety consistent with the items developed in this study [44]. Moreover, the questionnaire designed by Zarei covers eight dimensions, three of which are accessibility, physical environment, and physician’s consultation, as the dimensions in our questionnaire [45].

It should be noted that in some cases, although the names of the dimensions obtained from different studies may differ, most items are the same in the subset since selecting the names of dimensions is a matter of personal taste. Based on the results of the EFA test, the dimensions of the questionnaire (9 factors) explain about 73% of the total variance of service quality. The most important dimensions in explaining the variance were interactions (4 items) and efficiency (4 items), accounting for 21% of the total variance. The amount of the total reported variance (73%) in this study was higher than the declared standard level (60%) [43]. Similarly, in his study, Lee designed a questionnaire for health services, and the results indicated that the five dimensions of empathy, tangibility, safety, efficiency, and improvement of care explained about 70% of the total variance.

Furthermore, empathy (almost 49%) had the largest share of the total variance [35]. Also, the results of the study by Ogaji et al. indicated that a 3-component questionnaire explained 56.6% of the common variance of perceived quality of PHC in Nigeria [44]. Generally, although the classification of service quality dimensions is very important, there must be a consensus about the number of dimensions. However, all researchers confirm that service quality is a multidimensional issue with a complex structure [45].

The value of Cronbach’s alpha for all dimensions was above 0.7, and the value for the questionnaire was 0.82 indicating a good correlation between the items and internal consistency of the questionnaire and, therefore, the high reliability of the tool. In Lee’s study, all dimensions had Cronbach’s alpha coefficient above 0.8, higher than ours [35]. This index was also reported in Mosadeghrad and Sokhanvar’s study, where Cronbach’s alpha coefficient was 0.82 which is consistent with our study [36].

### Limitations

The study’s first limitation was budget and time constraints; we only selected three out of seven health networks, which might limit the generalizability of the results to other networks and health centers. The study was limited to health centers in Tehran province only, and the findings might not be fully applicable to other healthcare settings and environments.

## Conclusion

This study was conducted to develop a multi-dimensional questionnaire for measuring PHC quality in Iran. Based on the findings, the reliability and validity of the developed questionnaire were statistically significant; therefore, it can be used for evaluating the quality of primary care. This questionnaire can be considered as a standard tool for measuring the quality of PHC although researchers can further investigate and develop it with different samples of primary healthcare clients. In addition, the developed dimensions can be used in different languages or cultures, which requires cross-cultural validation studies.

### Electronic supplementary material

Below is the link to the electronic supplementary material.

Supplementary Material 1

## Data Availability

The datasets used and analyzed during the current study are available from the corresponding author upon reasonable request due to privacy.

## Abbreviations

PHC: Primary health care
WHO: World Health Organization
IUMS: Iran University of Medical Sciences
SDGs: Sustainable Development Goals
CVR: Content validity ratio
CVI: Content validity index
EFA: Exploratory factor analysis
KMO: Kaiser–Meyer–Olkin
