# Supplementary material for: Development and validation of primary health care quality assessment tool
Source: BMC Health Serv Res. 2023 Oct 26;23:1156. doi: 10.1186/s12913-023-10162-x (PMC10601108; doi:10.1186/s12913-023-10162-x)
Supplement: Supplementary file 1 — Supplementary Material 1 [file 12913_2023_10162_MOESM1_ESM.docx]

**Primary health care quality assessment tool (PHCQAT)**

**Dimensions and items**

Interactions: 19, 22-24

Efficiency: 25, 27-29

Timeliness: 20, 21, 26, and 30

Accuracy: 13-15

Consultation: 16-18

Tangibility: 1-4

Safety: 11 and 12

Accessibility: 7-10

Environment: 5 and 6

| **Primary health care quality assessment questionnaire (30-item)** | | | | | |
| --- | --- | --- | --- | --- | --- |
| **Items** | **Strongly agree** | **Agree** | **Not sure** | **Disagree** | **Strongly disagree** |
| 1: The health center was clean and tidy. |  |  |  |  |  |
| 2: The appearance and dress of the employees were neat and orderly. |  |  |  |  |  |
| 3: The amenities of the waiting room (TV, water cooler, chairs, magazines, etc.) were at the optimum level. |  |  |  |  |  |
| 4: This center has advanced equipment and facilities. |  |  |  |  |  |
| 5: The noise and crowding of the environment was not annoying. |  |  |  |  |  |
| 6: The temperature (hot and cold) of the environment was suitable. |  |  |  |  |  |
| 7: The hours and days of operation of the health center are such that you can easily visit it anytime. |  |  |  |  |  |
| 8: Access to the health center was easy. |  |  |  |  |  |
| 9: It is possible to receive medical advice from this center offline (e.g., via phone, website, etc.). |  |  |  |  |  |
| 10: If the doctor is absent in the health care center, someone else (such as a nurse) will handle your problem. |  |  |  |  |  |
| 11: In this center, a safe and comfortable environment is provided to receive services. |  |  |  |  |  |
| 12: Adequate measures are taken in this center to prevent the spread of infection. |  |  |  |  |  |
| 13: Doctors of this center do not make mistakes in their diagnosis. |  |  |  |  |  |
| 14: Nurses and other employees do not make mistakes in providing services. |  |  |  |  |  |
| 15: Doctors and employees have enough expertise and skills to provide service. |  |  |  |  |  |
| 16: In this center, timely measures and sufficient guidance are provided to relieve the patient from pain. |  |  |  |  |  |
| 17: Besides the visit, the doctor discussed ways to prevent other physical and mental diseases (e.g., diabetes, blood pressure, depression, anxiety, etc.). |  |  |  |  |  |
| 18: I received good advice from the doctor or nurse about a healthy lifestyle (e.g. healthy eating, exercise, etc.). |  |  |  |  |  |
| 19: The behavior of the employees (e.g., receptionist, security guard, and cashier) was good. |  |  |  |  |  |
| 20: Filing the case was easy and completed in the shortest possible time. |  |  |  |  |  |
| 21: The process of paying for the visit was easy and fast. |  |  |  |  |  |
| 22: The doctor and other employees were respectful and polite in providing services. |  |  |  |  |  |
| 23: The doctor and other employees answered my questions completely. |  |  |  |  |  |
| 24: The doctor's and other employees' words were clear and understandable. |  |  |  |  |  |
| 25: I visited the doctor on the expected day and hour. |  |  |  |  |  |
| 26: I did not wait long when I entered the health center to the doctor's room. |  |  |  |  |  |
| 27: The cost of a doctor's visit to this center is reasonable. |  |  |  |  |  |
| 28: The service I received at this center was worth paying for. |  |  |  |  |  |
| 29: The doctor and nurse were warned and reminded about the arbitrary use of medicine and other medical services without a prescription. |  |  |  |  |  |
| 30: the doctor and other staff tried their best to avoid wasting time. |  |  |  |  |  |
